# The development and implementation of a proficiency testing program for SARS-CoV-2 using dried tube specimens in resource-limited countries

**DOI:** 10.1186/s12879-024-09555-y

**Published:** 2024-06-27

**Authors:** Pius Lutaaya, Ocung Guido, Hasifah Nakato Ssentamu, George William Kasule, Mary Akumu, Jupiter Marina Kabahita, Bernard Bagaya, Kenneth Musisi, Denis Oola, Anitah Katuramu, Andrew Nsawotebba, Edgar Kigozi, Faith Nakazzi, Joel Kabugo Solomon, Isa Adam, Orena Beatrice, Joanita Namutebi, Brenda Ayebare, Abdunoor Nyombi, Charles Manyonge, Ademun Julius Patrick, Kangave Fredrick, Moses L Joloba

**Affiliations:** 1 National Tuberculosis Reference Laboratory/WHO Supranational Reference Laboratory, Kampala, Uganda; 2 Department of Immunology and Molecular Biology, College of Health Sciences, Makerere University, Kampala, Uganda

**Keywords:** External quality assessment, SARS-CoV-2, Proficiency testing, Dried tube specimens

## Abstract

**Introduction:**

When COVID-19 hit the world in 2019, an enhanced focus on diagnostic testing for SARS-CoV-2 was essential for a successful pandemic response. Testing laboratories stretched their capabilities for the new coronavirus by adopting different test methods. The necessity of having external quality assurance (EQA) mechanisms was even more critical due to this rapid expansion. However, there was a lack of experience in providing the necessary SARS-CoV-2 EQA materials, especially in locations with constrained resources.

**Objective:**

We aimed to create a PT (Proficiency testing) programme based on the Dried Tube Specimens (DTS) method that would be a practical option for molecular based SARS-CoV-2 EQA in Low- and Middle-Income Countries.

**Methods:**

Based on previous ISO/IEC 17043:2010 accreditation experiences and with assistance from the US Centers for Disease Control and Prevention, The Supranational Reference Laboratory of Uganda (adapted the DTS sample preparation method and completed a pilot EQA program between 2020 and 2021. Stability and panel validation testing was conducted on the designed materials before shipping to pilot participants in six African countries. Participants received a panel containing five SARS-CoV-2 DTS samples, transported at ambient conditions. Results submitted by participants were compared to validation results. Participants were graded as satisfactory (≥ 80%) or unsatisfactory (< 80%) and performance reports disseminated.

**Results:**

Our SARS-CoV-2 stability experiments showed that SARS-CoV-2 RNA was stable (-15 to -25 °C, 4 to 8 °C, (18 to 28 °C) room temperature and 35 to 38 °C) as well as DTS panels (4 to 8 °C, 18 to 28 °C, 35 to 38 °C and 45 °C) for a period of 4 weeks. The SARS-CoV-2 DTS panels were successfully piloted in 35 test sites from Zambia, Malawi, Mozambique, Nigeria, and Seychelles. The pilot results of the participants showed good accuracy, with an average of 86% (30/35) concordance with the original SARS CoV-2 expectations.

**Conclusion:**

The SARS-CoV-2 DTS PT panel is reliable, stable at ambient temperature, simple to prepare and requires minimal resources.

## Introduction

Coronavirus disease (COVID-19) is an infectious disease caused by the severe acute respiratory syndrome coronavirus 2 (SARS-CoV-2) [1]. Due to its rapid and efficient mode of transmission, COVID-19 was declared as a pandemic and compelled adaptation of various testing platforms to meet laboratory diagnostic needs [2]. Timely and accurate diagnostic testing for SARS-CoV-2 is a critical component to the overall prevention and control strategy for COVID-19 [3]. Several diagnostic techniques for SARS-CoV-2 virus detection are available. These include (1) detection of viral RNA, through manual or automated nucleic acid amplification tests (NAAT), such as real-time reverse-transcription polymerase chain reaction (RT-PCR), (2) detection of viral antigens through immunodiagnostic techniques, such as lateral flow assays (LFAs), commonly called rapid diagnostic tests or Ag-RDTs and (3) detection of host antibodies through serological techniques, such as LFAs, enzyme-linked immunosorbent assays (ELISAs), or chemiluminescent immunoassays (CLIAs) [4]. While these tests have received emergency use authorization. the World Health Organization (WHO) consequently encourages testing facilities to participate in external quality assessment (EQA) schemes for this novel virus [5].

In 2020 and 2021 Uganda accredited 21 SARS-CoV-2 testing sites which included government and private testing facilities at national and regional levels, and at border points [6]. The SARS-CoV-2 laboratory testing network began to expand, calling for the establishment of mechanisms to monitor and ensure the quality of testing in the growing network. With rare and novel diseases including COVID-19, the identification of reliable and consistent proficiency testing (PT) providers remain a challenge. Participating in an EQA program help laboratories identify shifts and trends that would otherwise have gone unnoticed and verify the reliability of their testing results [7–9]. The term external quality assessment (EQA) is used to describe a method that allows for comparison of a laboratory’s testing to a source outside the laboratory. The term EQA is sometimes used interchangeably with proficiency testing; however, EQA can also be carried out using other processes like onsite support and blinded rechecking [10].While varieties of SARS-CoV-2 diagnostic tests are being used globally to test patients suspected of COVID-19 disease, there are insufficient EQA programs, especially in Low and Middle-income Countries. Most of the limited number of SARS-CoV-2 EQA panels currently available in resource-limited settings are procured from commercial international providers.

To address these challenges, we adapted a method used for preparing DTS PT panels for molecular TB for SARS-CoV-2. The Supranational Reference Laboratory of Uganda (SRL-Uganda), previously established an accredited ISO 17043:2010 PT program and readily began the adaptation of DTS technology to prepare a SARS-CoV-2 PT panel preparation procedure. The process involved several steps, including stability testing, validation, and pilot testing of prepared DTS samples. Here, we present the results from the pilot PT panel for molecular detection of SARS-CoV-2 developed by SRL-Uganda with technical support from the U.S. Centers for Disease Control and Prevention (CDC) using the DTS methodology, originally deployed in HIV rapid test PT programs [11, 12].

## Materials and methods

### Virus stock material development and stability

Initial evaluation and optimization of a SARS-CoV-2 PT panel started with finding suitable materials and determining the stability of the materials. Nucleic acids (RNA) were isolated and purified from upper respiratory clinical specimens (such as nasal, mid-turbinate, nasopharyngeal, oropharyngeal swab specimens and nasopharyngeal wash/aspirate or nasal aspirate specimens). Uncharacterized, clinical, known-positive COVID-19 samples, with a cycle threshold (Ct) less than 25 when the N2 gene was amplified using RT-PCR, were selected for extraction. Upon pooling, the final Ct value of 14.7 was obtained. Extraction of SARS-CoV-2 RNA was performed according to CDC 2019-Novel Coronavirus (2019-nCoV) Real-Time RT-PCR Diagnostic Panel [13]. The extracted SARS-CoV-2 RNA was pooled to make viral stock of working volume 10,000 µl. Aliquots were prepared by transferring 100 µl of viral RNA into uncapped 2 ml screw-cap Sarstedt cryogenic vials. Prior to testing, the tubes were capped and stored at (-15 to -25 °C, 4 to 8 °C, (18 to 28 °C) room temperature and 35 to 38 °C. Aliquots from each storage temperature condition were tested in triplicate on the Applied Biosystems TM ABI 7500 Real-Time PCR system using described CDC 2019-Novel Coronavirus (2019-nCoV) Real-Time RT-PCR Diagnostic Panel [13] at weeks 1, 2, 3, and 4 and mean Cycle thresholds for the N2 gene were compared across storage conditions and time points.

### Polymerase chain reaction (PCR) assays

Extraction of RNA was completed per manufacturer’s instructions using the QIAamp^®^ Viral RNA Mini Kit (Cat No. 52906). Briefly, the purified nucleic acid (5 µl) was reverse transcribed using COVID-19 Nucleic Acid RT- PCR Test Kit (catalog # A15299) (15 µl PCR reaction solution) into complimentary DNA (cDNA) which is then subsequently amplified in Applied Biosystems™ 7500 Real-Time PCR system. In this process, the probe anneals to the N2 target sequence located between the forward and reverse primers. During the extension phase of the PCR cycle, the 5’ nuclease activity of Taq polymerase degrades the probe, causing the reporter dye to separate from the quencher dye, generating a fluorescent signal. With each cycle, additional reporter dye molecules are cleaved from their respective probes, increasing the fluorescence intensity. Fluorescence intensity is monitored at each PCR cycle.

### Preparation of DTS and stability testing

Following extracted RNA optimization and stability experiments, DTS were prepared based on the protocol described in previous studies [11, 14]. The viral stock was diluted by adding 0.5 ml into 9.5 ml of molecular grade water (Catalog#BP561-1, Fisher) making 1:10 dilution, premixed with 0.1% (v/v) blue liquid food dye to each dilution, and then transferring 100 µL of each stock dilution into five 2 mL cryovials [15]. The food dye allows for visualization of the pellet at the bottom of the tube after sample drying. Dried Tube Specimen (DTS) samples were prepared by aliquoting 100 µl of diluted RNA viral stock into uncapped 2 ml screw-cap cryogenic vials and then leaving samples open to dry in a Class II BSC for 10 days. The BSC was left running during the drying period to protect DTS from contamination and to speed drying. The DTS were then capped and stored at 4 to 8 °C, 18 to 28 °C, 35 to 38 °C and 45 °C. Stability studies were completed by testing triplicate sets across multiple time points. Dried tube specimen samples were rehydrated by adding 600 µl of RNA grade water (Catalog#BP561-1, Fisher) before testing. The DTS were mixed by vortexing for 15 s and then incubated at room temperature for 4 h to allow for solubilization. Rehydrated DTS were re-extracted according to the procedure described by (CDC 2019-Novel Coronavirus (2019-nCoV) Real-Time RT-PCR Diagnostic Panel [13].). One triplicate set was rehydrated at week 0 (baseline) and one set from each of the four storage conditions was rehydrated at weeks 1, 2, 3, and 4 and analyzed using Applied Biosystems™ 7500 Real-Time PCR equipment to obtain the Ct values. Mean Ct values were compared across storage conditions and time points to assess the stability of SARS-CoV-2 DTS samples.

### Field validation exercise

The homogeneity of prepared panels was maintained through internal quality control procedures. Similar batches of extracted SARS-CoV-2 RNA suspension were prepared as described above. A validation study was conducted to assess the performance of the SARS-CoV-2 DTS PT panel with various reagents and equipment. The panels were sent to 25 enrolled, certified testing sites (13 in Kenya and 12 in Uganda). The locations of enrolled testing sites were diverse and spread out across both countries. Panels were freshly made with unique compositions of 3 positives and 2 negative samples and coded from SARS-CoV-2 X-1 to X-5. Simple instructions and reporting forms were developed to capture testing data (SARS-CoV-2 detection results, Ct values, types of equipment, reagent and viral genome targets). This to enabled collection of more information concerning the stability and accuracy of the prepared DTS samples.

### Pilot study

A pilot test was conducted to gauge how well the SARS-CoV-2 DTS PT panel performs under real-world circumstances in an international setting. The survey panels were sent to 56 enrolled testing sites from 6 countries: Zambia (29) Malawi (5) Mozambique (4) Nigeria (10) Namibia (4) and Seychelles (4). The panels were made up of three SARS-CoV-2 positive and two SARS-CoV-2 negative DTS samples. All panels were transported at ambient temperature. Straight-forward instructions and reporting forms were included (SARS-CoV-2 detection result, Ct values, types of equipment, reagent and genome targets) with the panel to instruct participants on rehydration of DTS samples and for result submission. Sites were asked to treat and test rehydrated DTS samples like clinical samples using routine molecular assay workflows and to report results as mentioned above. Data analysis was performed using MS Excel to determine means, standard deviations, and delta Ct values.

## Results

### Homogeneity and stability of RNA viral stock material

**Fig. 1:**
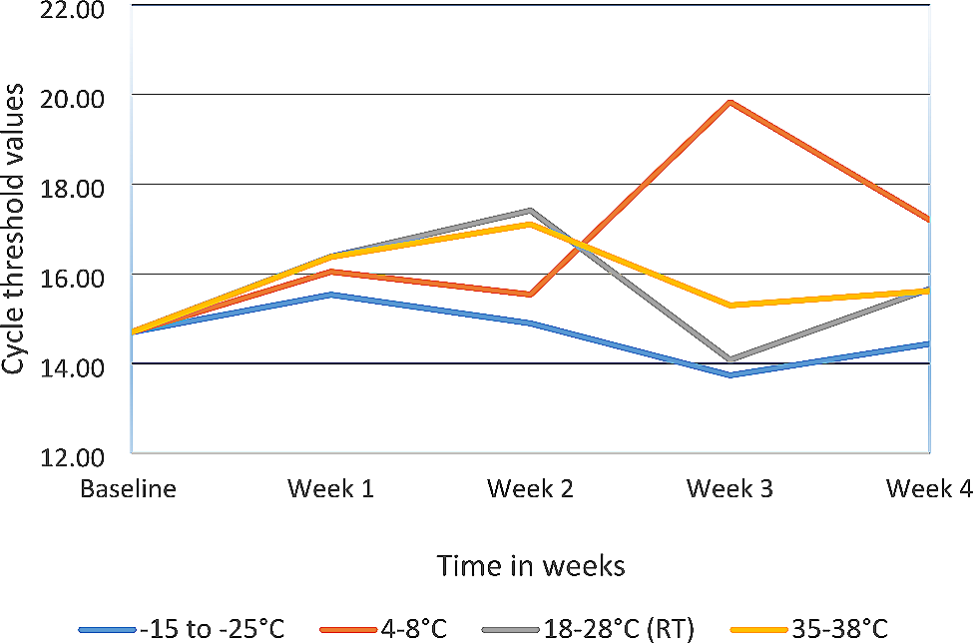
Thermal stability of SARS-CoV-2 RNA concentration at different temperatures − 15- -25 °C, 4–8 °C, 18–28 °C (RT), and 35–38 °C over a period of 4 weeks as tested using Applied BiosystemsTM ABI 7500 Real-Time PCR system

**Fig. 2:**
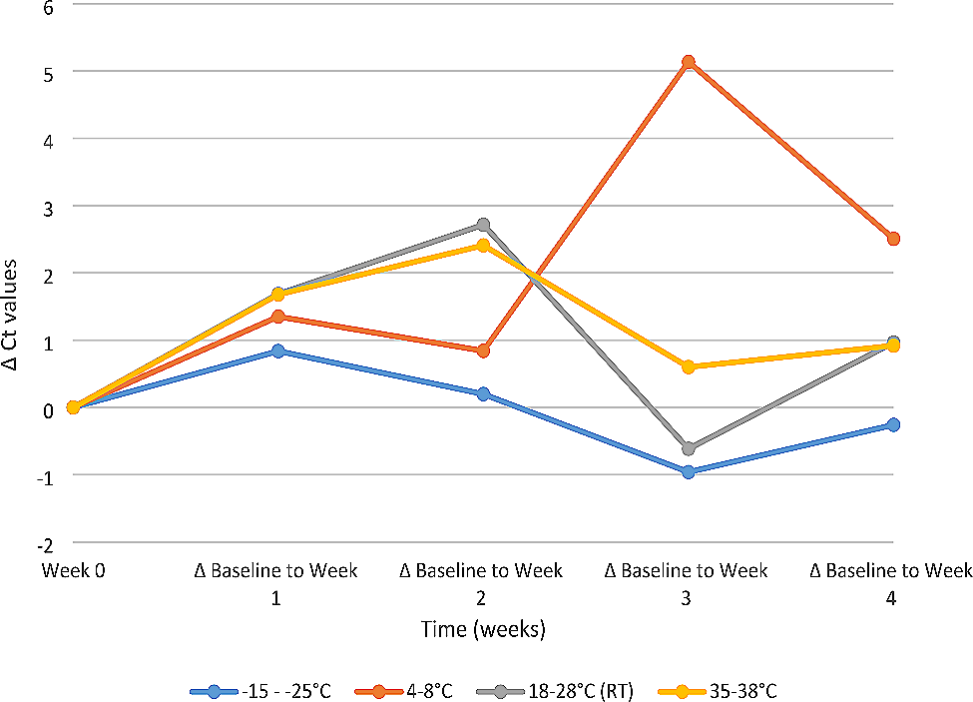
Evaluation of the change of Ct values from baseline (week 0) of SARS-CoV-2 RNA concentration at different temperatures − 15 - -25 °C, 4–8 °C, 18–28 °C (RT), and 35–38 °C over a period of 4 weeks as tested using Applied BiosystemsTM ABI 7500 Real-Time PCR system

Analysis of the initial extracted SAR-CoV-2 viral RNA stock concentration stored at the different temperatures yielded comparable Ct values as seen in the Fig. 1. There was a slight increase in mean Ct values at all the temperatures when comparing the baseline Ct value and forth week. As shown in Fig. 2, the findings for the temperature range of 4–8 °C during the third week testing were 5 Ct values greater than the baseline. An average rise of 3 Ct values was observed until week four at − 15- -25 °C, 4–8 °C, 18–28 °C (RT), and 35–38 °C.

### Homogeneity and stability of SARS-CoV-2 DTS panels

**Fig. 3:**
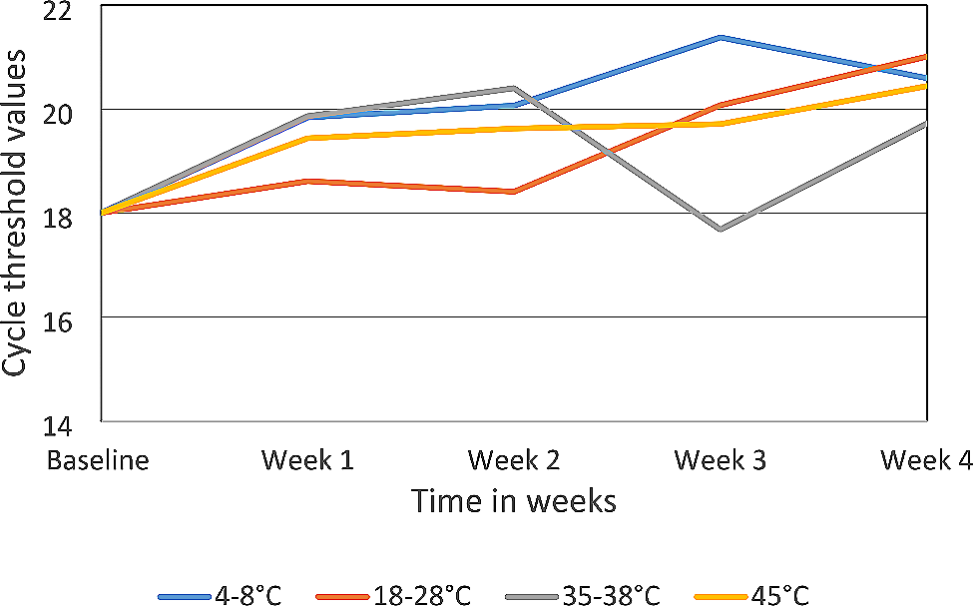
Thermal stability of SARS-CoV-2 DTS at different temperatures 4–8 °C, 18–28 °C, 35–38 °C, and 45 °C as tested over a period of 4 weeks using Applied BiosystemsTM ABI 7500 Real-Time PCR system

**Fig. 4:**
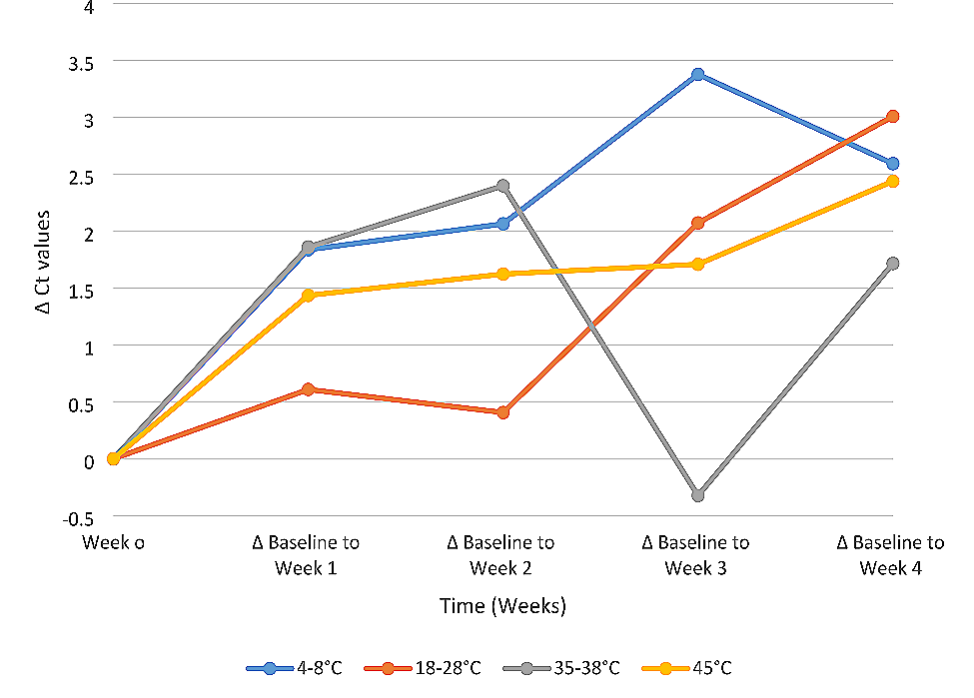
Assessment of the change in Ct values from baseline (week 0) of SARS-CoV-DTS concentration at various temperatures over a period of 4 weeks: 4–8 °C, 18–28 °C (RT),35–38 °C and 45° C as tested using Applied BiosystemsTM ABI 7500 Real-Time PCR system

The mean Ct values rise linearly up to week 4, regardless of storage temperature. According to Fig. 3, the highest Ct values were seen at week 3 for storage temperatures between 4 and 8 °C and the lowest values were seen at the same time for temperatures between 35 and 38 °C. At all the storage conditions, findings revealed an increase of roughly 3 Ct values at points across the four weeks of testing as depicted in Fig. 4.

### Field validation of SAR CoV-2 DTS panel

Among the 25 certified testing sites (13 in Kenya and 12 in Uganda) enrolled in the field validation panel, eleven from Uganda and nine from Kenya (78%) submitted results by the closing date. Panel scoring was qualitative and participant’s performance was determined by comparing submitted SARS-CoV-2 detection results to expected results established during homogeneity testing prior to shipment. The average total score for each panel was 100% Table 1.

**Table 1:** The expected outcomes versus participant’s consensus SARS-CoV-2 DTS panel outcome

Panel ID	Expected Result	Participant Result	Participant Score
SARS-CoV-2 X-1	SARS-CoV-2 Not Detected	SARS-CoV-2 Not Detected	100%
SARS-CoV-2 X-2	SARS-CoV-2 Not Detected	SARS-CoV-2 Not Detected	100%
SARS-CoV-2 X-3	SARS-CoV-2 Detected	SARS-CoV-2 Detected	100%
SARS-CoV-2 X-4	SARS-CoV-2 Detected	SARS-CoV-2 Detected	100%
SARS-CoV-2 X-5	SARS-CoV-2 Detected	SARS-CoV-2 Detected	100%

The SARS-CoV-2 PCR detection kits used include GeneXpert^®^ + Xpert^®^ Xpress SARS-CoV-2 (Cepheid Europe SAS, Maurens-Scopont, France) (10), Abbott (3), Qiagen (1), TaqPath COVID assay (1), Standard N nCoV (1), Virokey (1), Altona (1) and Real star assay (1). Facilities from Uganda used a variety of PCR reagents and equipment and whereas Kenyan counterparts used only Cepheid GeneXpert Xpert Xpress SARS-CoV-2 as seen in the Table 2 below.

**Table 2:**
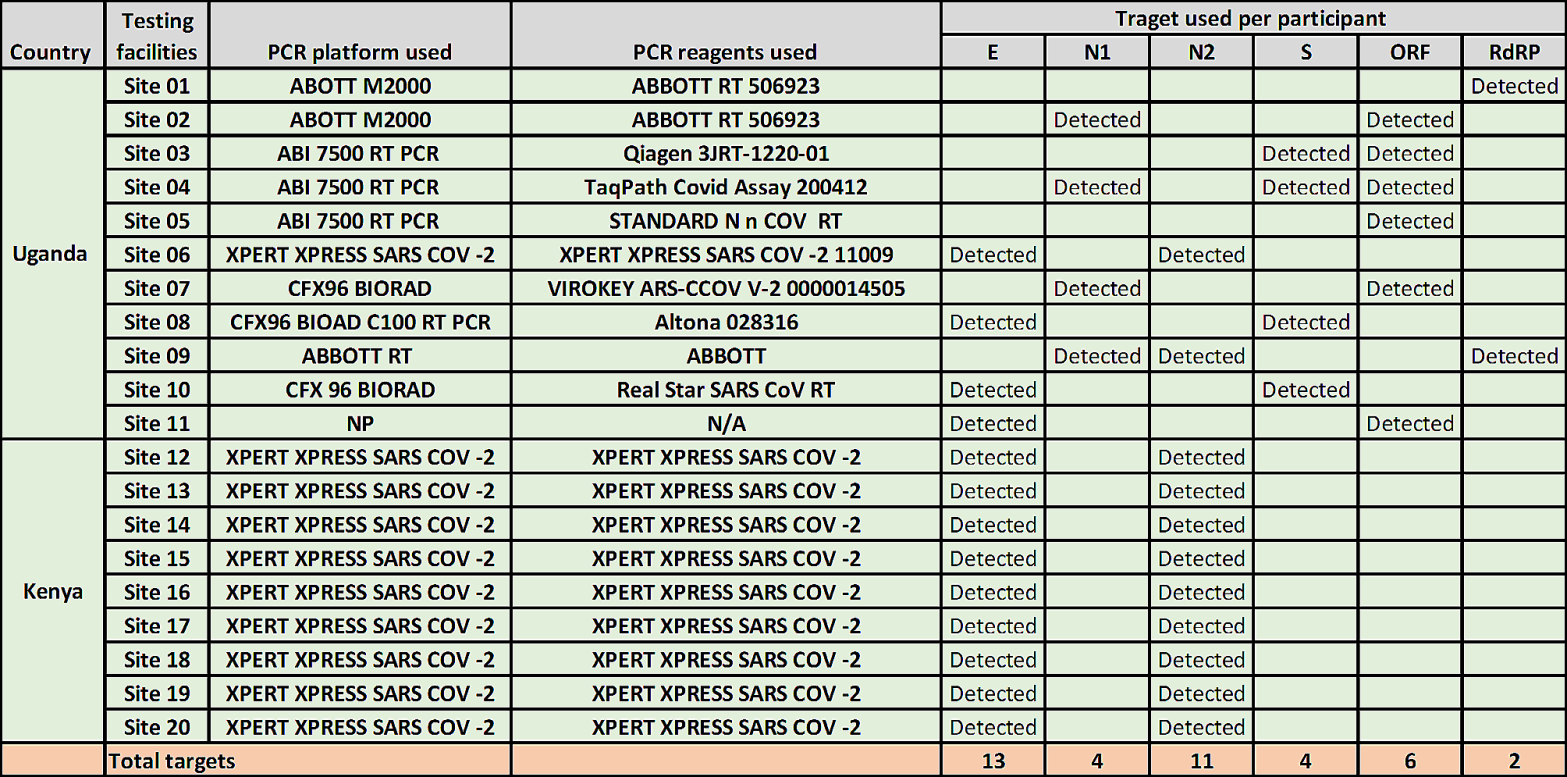
PCR testing platforms and SARS-CoV-2 gene targets tested by participants from Uganda and Kenya

### Pilot study

**Fig. 5:**
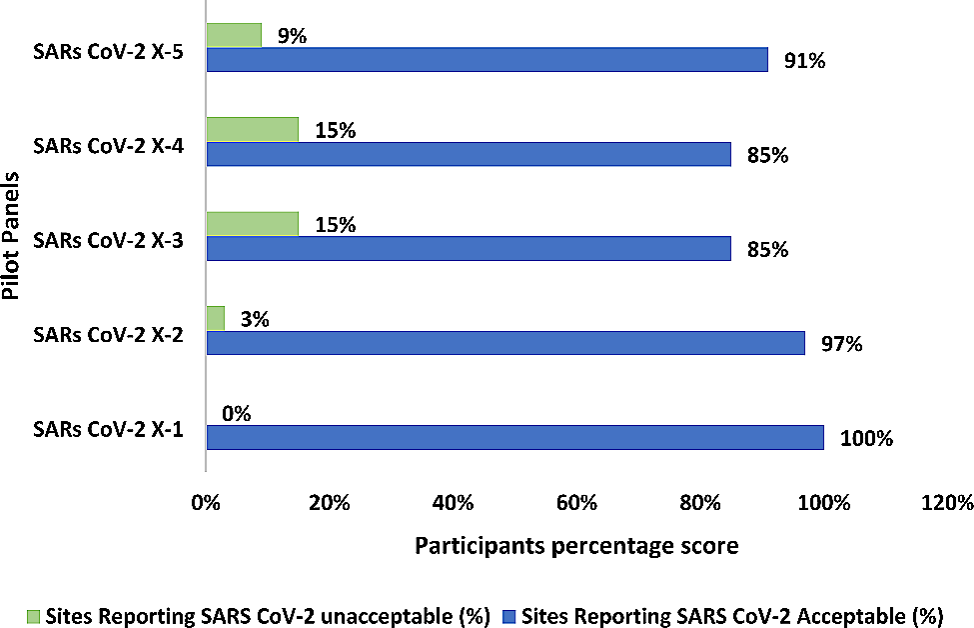
Individual SARS-CoV-2 PT sample concordance from 35 testing sites participating in the pilot study

In the pilot study, there were 56 sites enrolled, and 35 (63%) of them were able to submit their results by the deadline. The proportion of participating sites with satisfactory results was 30/35(86%). Twenty-five participants scored 100%, five scored 80%, three scored 60%, and two scored 40%. The participation rate breakdown by country was as follows: Zambia − 20/29 (69%), Malawi − 3/5 (60%), Mozambique − 4/4 (100%), Nigeria − 4 /10 (40%), Seychelles − 4/4 (100%) and Namibia-0/4 (0%) The individual sample concordance of results reported by testing sites with expected result was: SARS-CoV-2 X-1 (100%), SARS-CoV-2 X-2 (97%), SARS-CoV-2 X-3 and SARS-CoV-2 X-4 (85%), and SARS-CoV-2 X-5 (91%) as reflected in Fig. 5.

### SARS CoV-2 gene targets by sites

**Fig. 6:**
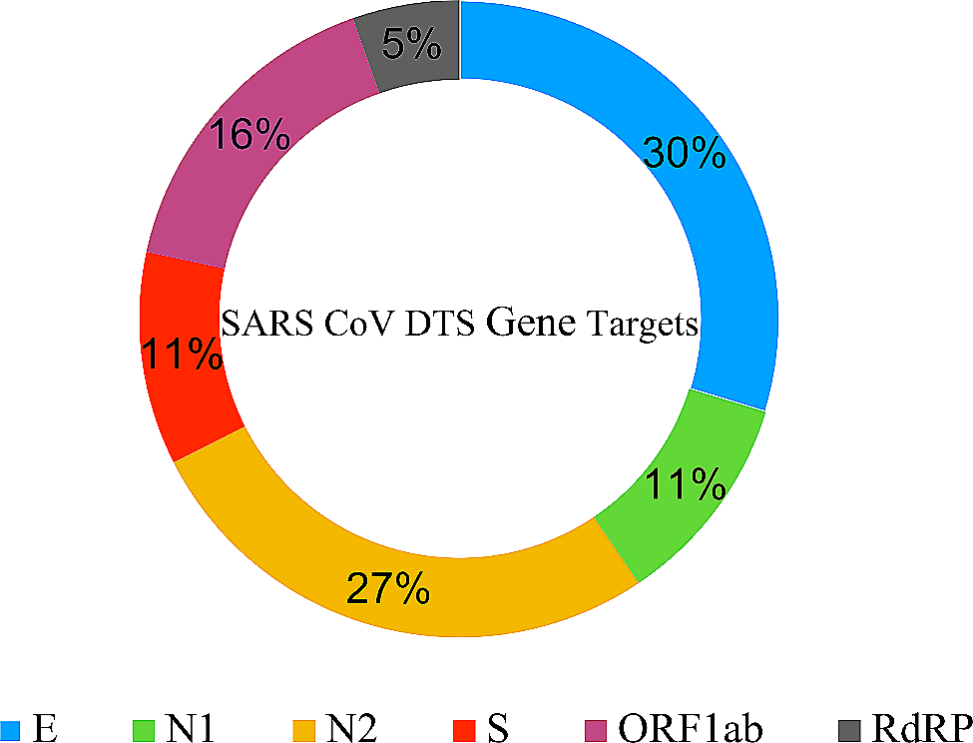
Gene targets detected by different sites in the SARS-CoV-2 DTS pilot survey. By target, commercial kits showed similar performance and reported comparable SARS CoV-2 results

The different commercial kits used by sites amplified five unique targets in the SARS-CoV‐2 genome, including: nucleocapsid (N) gene, envelope (E) gene, Spike protein (S) gene, RNA‐dependent RNA polymerase (RdRP) gene large open reading frame (ORF1ab) as shown.

in Fig. 6 by target. Commercial kits used by sites showed similar performance and reported comparable SARS CoV-2 results in the pilot.

## Discussion

There was a significant demand for the establishment of EQA mechanisms within diagnostic laboratories during the COVID-19 pandemic to ensure continuous quality improvement and quality testing of patient samples. Available programs were costly and were accompanied by logistical challenges, rendering them unfeasible in locations with low resources. The long-term practice in providing PT materials through annual programs and the recent development of a DTS PT panel for HIV-1 serology and tuberculosis described by Parekh and colleagues in 2009 [11] and by Ramos and colleagues 2013 [14] triggered the development and adaptation of DTS for SARS-CoV-2 PT detailed in this work. Clinical samples from SARS-CoV-2-infected patients do contain viral RNA at concentrations sufficient to enable the creation of stable and accurate DTS PT panels. The evaluation panel launched at the perfect time to support recently created molecular laboratories performing SARS-CoV-2 PCR testing and enabled these facilities to be supported with PT materials prepared locally. The SARS-CoV-2 DTS panel has a number of benefits. (1) It is inexpensive to produce and ship. (2) Dried samples are unable to leak and offer a higher level of biosafety protection during transportation. (3) Dried Tube Specimen samples are stable at a wide range of temperatures and (4) require minute volumes of RNA stock to prepare.

The SARS-CoV-2 RNA from clinical specimen remained stable when stored at -15 to -25 °C, 4 to 8 °C, (18 to 28 °C) room temperature and 35 to 38 °C for 4 weeks as shown by the Ct values in Fig. 1. The observed fluctuating Ct values at storage temperature 4 to 8 °C at 3 and 4 week as compared to the baseline Ct value in Fig. 2 can be attributed to human error during sample preparation. Extracted viral RNA was stable due to the chemical components (Sodium azide, DNA/RNA shield) used in extraction kits that prevents RNases [16, 17].

As shown in Fig. 3, dried tube specimens were stable at the temperatures used in this study’s experiments. Compared to the baseline value shown in Fig. 4, the Ct values at all storage temperatures varied between 0 and 3 Ct values. RNases are rendered inactive by drying, which is the cause of this stability in the DTS [18]. The DTS is more stable as compared to the liquid matrix. The ambient temperature is suitable for shipping panels to the testing laboratories as demonstrated in the stability study. This adds to the earlier finding detailing stability and feasibility of DTS shipped at ambient temperature as described by Bharat Parekh and colleagues 2009 and Kyle DeGruy and colleagues 2020 [11, 15].

We verified the prepared SARS-CoV-2 DTS using external mechanisms in order to assess the performance on various testing platforms and with a variety of reagents used to detect SARS-CoV-2. This was done due to the limited equipment and reagents in-house at the time. All sites in the validation exercise achieved 100% concordance in comparison with expected results. Participating sites used a variety of PCR reagent targeting diverse sections of the viral genome. Targets included: RdRP, N1, N2, ORF1ab, S and E. It was observed that different testing platforms yielded similar results for SAR-CoV-2 target detection. These findings add to the study done by Buchta and colleagues 2020 where 91/101 (90.1%) PT participants testing a large varieties of RT-PCT assays correctly identified the three positive samples [19].

During the pilot, the highest concordance was observed in the Negative samples (SAR-CoV-2 -X-1 and SAR-CoV-2 -X-2). False negative discordance were observed in positive sample SAR-CoV-2 -X-5, SAR-CoV-2 -X-3 and SAR-CoV-2 -X-4. The reason for reporting false negative results is not yet known. However, some studies have described causes of false negatives such as thermal inactivation of SARS-CoV-2, targets of SARS-CoV-2 genome, PCR method sensitivity, co-infection with other viruses, viral load, optimal time among others [20, 21]. The high proportion (86%) of testing sites scoring satisfactory demonstrated the preparation of quality panels. Its worthy noting that there is no substantive correlation between the false negative results obtained from participants and the testing platform.

### Limitation

The cardinal challenge in producing the SARS-CoV-2 DTS was an insufficient number of clinical samples and shift in the sample volume during the pandemic. Numerous samples needed to be extracted to obtain optimal volumes and concentrations of SARS-CoV-2 RNA to prepare PT panels. Another drawback encountered during the pilot PT round was low consensus (86%) with the expected results, primarily due to testing sites reporting false-negative SARS-CoV-2 results. There was a low result return rate which may be due to this being the first time COVID − 19 testing sites were asked to participate in PT and shipping delays and increased custom clearance time resulting from operating during a pandemic may have played a role in successful delivery of panels to testing sites. The stability of prepared SARS-CoV-2 DTS was not tested beyond 4 weeks, and developed panel was limited to use in PCR COVID-19 based assays.

## Conclusion

The goal of this endeavor was to develop a simple procedure to prepare a reliable, accurate, and stable PT panel for SARS-CoV-2 using minimal resources. The data presented above demonstrates successful development of a PT panel meeting the above parameters using the Dried Tube Specimen method. This procedure can be replicated in other labs to monitor the performance and aid in ensuring quality testing of molecular SARS-CoV-2 testing networks in other low to middle income countries.

## Data Availability

All relevant data are within the paper and its supporting information files.
